# Cycloartanes from *Euphorbia aellenii* Rech. f. and their Antiproliferative Activity

**Published:** 2011

**Authors:** Abdul Majid Ayatollahi, Mustafa Ghanadian, Suleiman Afsharypuor, M. Ahmad Mesaik, Omer Mohamed Abdalla, Mohsen Shahlaei, Gholamhossein Farzandi, Hamid Mostafavi

**Affiliations:** a School of Pharmacy and Phytochemistry Research Center, Shahid Beheshti University of Medical Sciences, Tehran, Iran.; b Pharmaceutical Sciences Research Center, Isfahan University of Medical Sciences, I.R. Iran.; c Department of Pharmacognosy, Faculty of Pharmacy and Pharmaceutical Sciences, Isfahan University of Medical Sciences, Isfahan, Iran.; d Dr. Panjwani Center for Molecular Medicine and Drug Research, International Center for Chemical and Biological Sciences, University of Karachi, Karachi-75270, Pakistan.; e Department of Medicinal Chemistry, Faculty of Pharmacy, Kermanshah University of Medical Sciences, Kermanshah, Iran.; f Welsh School of Pharmacy Centre for Socioeconomic Research, Cardiff University, UK.

**Keywords:** *Euphorbia aellenii*, Proliferation assay, Cycloartanes, Structure-activity relationship (SAR), Protein kinase C (PKC)

## Abstract

The cytotoxic chloroform fraction of *Euphorbia aellenii *afforded two cycloartane type triterpenes-cycloart-25-en-3*β*,24-diol (1) and 24-methylene-cycloartan-3β-ol (2)-for the first time from this plant. Preparation of cycloartane derivatives, 3*β*, 24-O-diacetyl-cycloart-25-en as compound 3 and 3*β*-O-acetyl-24-methylene-cycloartan (4) were conducted by acetylating of 1 and 2, respectively. The structures of the isolated compounds were elucidated by spectroscopic methods and their activities evaluated by proliferation assay on human peripheral blood lymphocytes (PBLs). Comparing the results suggested that anti-proliferation effect of these compounds on PBLs might be due to the presence of free 3-OH group while masking the free OH groups by acetylation, could induce proliferation activity.

## Introduction

**Figure 1:**
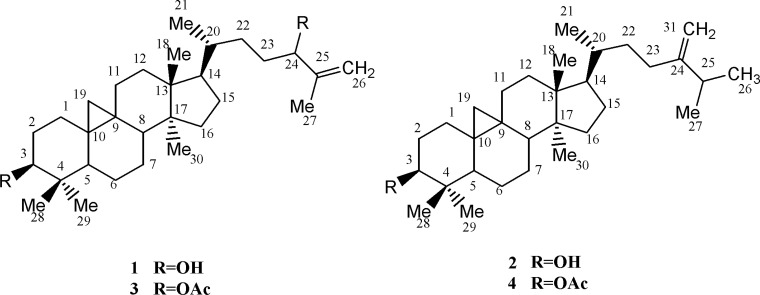
Cycloartanes from *E. aellennii *and their acetylated derivatives (1-4).

Cycloartanes (9, 19-Cyclolanostanes) are one of the main tetra-cyclic triterpene skeletons including the side chain and characteristic cyclopropane ring (1). They are the major key intermediates in the phytosterols biosynthesis (1). In addition, these compounds are used as specific chemotaxonomic markers in *Euphorbia *genus, which comprises well over 2000 species in tropical and temperate zones of asia and other parts of the world. In iran, 70 species are reported, 17 of which are endemic (2). Within our recent study on triterpenoids, present work describes the isolation and structure elucidation of two cycloartane type triterpenes from *Euphorbia aellenii *Rech. f. (Figure 1) along with their anti-proliferative activity on human peripheral lymphocytes and their structure-function studies on protein kinase C (PKC) that plays an important role, as an early event, in T-cell activation (3). 

## Experimental

*General*

Column chromatographie s (CC) were run silica gel 63-200 μm, LiChroprep® Si 60 (25-40 μm) and RP-18 (40-63 μm, Merck). The size exclusion chromatography (SEC) was rendered on sephadex (LH-20, Sigma-Aldrich) and recycling preparative HPLC on LC-908 (Hitachi Company, Japan), equipped with YMC Pack-Sil column (250*20 mm i.d). The NMR spectra were recorded on Bruker AV-300 (^1^H), AV- 600 (^13^C) and HR-ESI-MS on Waters Q-TOF Micro YA019 mass spectrometer.

*Plant material*

The aerial flowering parts of *Euphorbia aellenii *Rich. F. (euphorbiaceae) were collected from populations growing in Galil-e-Shirvan (Iran) and identified by Dr. Yasamin Naseh, herbaceous sciences research center at ferdowsi university of mashhad. A herbarium specimen No. 2024 is preserved in the herbarium of the faculty of pharmacy, isfahan university of medical sciences (Iran). 

*Isolation of cycloartanes*

The air-dried powdered plant (7 Kg) was macerated for four days with MeOH (20 L×3), at room temperature. Filtration and *in-vacu*o evaporation resulted in a green gum (500 g), which was partitioned between methanol and *n*-hexane. The defatted methanolic extract was concentrated, dissolved in water, and extracted sequentially with chloroform, ethyl acetate, *n*-butanol, and water. The resulted fractions (Fr._1_-Fr._4_) were compared *in-vitro *for cytotoxic activity (4), showing LD_50_ value of 177.06 (96.90-255.61) and 770.66 (670.06 -916.40) μg mL^− 1^ for Fr._1_ and Fr._2_, respectively while other fractions (Fr._3_ and Fr._4_) were marked as non-cytotoxic. The most active fraction (Fr._1_, 240 g) was subjected to silica gel column, using Hexane/CHCl_3_ (0→100), to render several fractions (Fr._1a_-Fr._1f_). Then, Fr._1b_ was eluted with Hexan/CHCl3 1:1 and chromatographed on silica gel (Hexane/EtOAc, 0→50) to come up with ten subfractions. Finally, 1 g of the fraction was eluted with Hexane/EtOAc 9:1, purified on recycling HPLC (Hexane/EtOAc 6:4, 4.0 mL/min), and consequently yielded two pure compounds: 1 (20 mg, t_R _200 min) and 2 (10 mg, t_R_ 220 min). 

*Preparation of cycloartane derivatives*

A mixture of cycloartane type compound (10 mg), acetic anhydride (1 mL) and pyridine (0.5 mL) were stirred at room temperature for three days. The final point of reaction was controlled by TLC and the reaction mixture was poured over ice to decompose the remaining acetic anhydride. Thereafter, suspension was extracted twice with ethyl acetate (2:1 v/v), and evaporated *in-vacuo *to yield acetylated products (3 and 4), which was confirmed by Mass and H-NMR spectra (5).

*Brine shrimp (artemia salina) cytotoxicity*

Shrimp eggs were added to a specific tank containing artificial seawater, hatched within two days and transferred to sample vials (1000, 100 and 10 μg/mL). 24 h later, keeping vials under illumination, the surviving shrimps were counted to obtain LC_50_ and 95% confident intervals (4). 

*Proliferation assay*

Peripheral human blood lymphocytes were incubated with different concentrations of the test compounds (0.5, 5, and 50 μg/mL (in triplicates) in supplemented RPMI-1640 along with phytohemagglutinin (PHA) at 37ºC in CO_2_ environment for 72 h. Further incubation for 18 h after the addition of thymidine [^3^H] (Amersham, UK) was done and cells were harvested using cell harvester (Innotech Dottikon, Switzerland). Finally, proliferation level was determined by the radioactivity count as CPM recorded from the Beta-scintillation counter (Beckman coulter, LS 6500, Fullerton, CA, USA) (6).

*Docking methodology*

Using the crystal structure of protein kinase C (PKC), retrieved from the protein data bank (PDB code: 1zrz), compounds (1-4) were selected for docking process and optimized by Polak-Ribiere conjugate gradient algorithm and AM1 semi empirical method, implemented in HyperChem. The optimized structures were used as the input of auto dock tools and the partial charges of atoms were calculated using gasteiger-marsili procedure (7). Merging non-polar hydrogens, rotatable bonds were assigned; after removing the heteroatom including water molecules from protein, all missing hydrogens were added. Thereafter, determining Kollman united atom charges (8), non-polar hydrogens were merged to their corresponding carbons and as a final processing part in protein preparation, desolvation parameters were assigned to each atom. Using auto Grid tool, the grid maps (one for each atom type in the ligand, and one for electrostatic interactions) were constructed adequately large to include the active site of protein as well as significant regions of the surrounding surface. In all the cases, a grid map of 60 points in each Cartesian direction apart from a grid-point spacing of 0.375 A° (a quarter of the carbon-carbon single bond) were generated. By the ligand location in the complex, the maps were centered on the ligand’s binding site, searching favorable interactions with the functional groups. Based on lamarckian genetic algorithm (9), using the pseudo-solis and wets local search method (10), Auto Dock Tools were employed to produce both grid and docking parameter files *i.e. *.gpf and .dpf files. Applying 2.0 A° clustering tolerance to construct clusters of the closest compounds, the initial coordinates of the ligand were used as the reference structure. In addition, for the internal validation phase, ligand structure (corresponding HETATM and CONECT records) was extracted from the pdb file of CDK_2_ (2BTS). Later, assigning bond orders, missed hydrogens were added and a short minimization (100 steepest descent steps were taken, using MM^+^ force field with a gradient convergence value of 0.05 Kcal/mol A°) to release any internal strain. At last, docking results (PKC-ligand complexes) were visualized using VMD1.8.6 (11). 

*Statistical analysis*

The IC_50_ values were calculated using Excel based program and reported as mean ± SD of the mean. Significance was attributed to p-values (p < 0.05) and the probability values obtained by the student t-test between the sample and control data. 

## Results and Discussion

Compound 1, white crystals with mp 180-184°C, showed the molecular formula of C_30_H_50_O_2_ based on positive EI-HR-MS m/z 442.3784 (calc. for C_30_H_50_O_2_: 442.3811, Δ 6.10 ppm) ), in accordance with the number and the multiplicity of ^13^C-NMR spectra (Table 1). 

**Table 1:** 13C-NMR data for the cycloartanes (1 and 2)**.**

^13^C	1	2
1	31.66t	31.97t
2	30.45t	30.38t
3	78.89d	78.85d
4	40.48s	40.49s
5	47.19d	47.1d
6	21.14t	21.14t
7	28.12t	28.17t
8	47.99d	48.02d
9	20.41s	19.98d
10	26.15s	25.78s
11	26.04s	26.04t
12	35.61t	32.88t
13	45.29s	45.29s
14	48.8s	48.81s
15	32.02t	34.95t
16	26.55t	26.46t
17	52.26d	52.26d
18	18.03q	18.07q
19	29.89t	29.93t
20	36.01d	36.35d
21	18.37q	18.23q
22	31.53t	35.57t
23	28.12d	31.31t
24	76.74d	156.96s
25	149.77s	33.8s
26	111.33t	22.02t
27	17.27q	19.34q
28	19.36q	18.31q
29	25.48q	14.03q
30	14.02q	25.45q
31	-	105.91t

^a^chemical shifts are given in ppm

**Figure 2:**
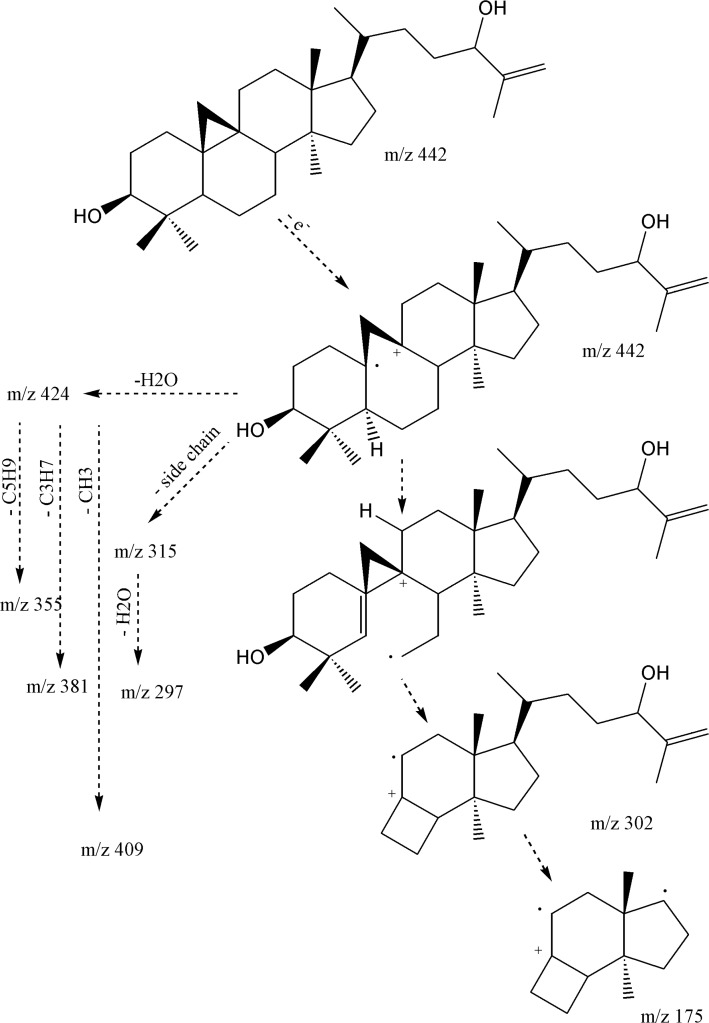
EI-Mass fragmentation pattern of cycloart-25-en-3β,24-diol

The IR spectrum confirmed presence of hydroxyl group (3373 cm^-1^), double bond absorption (1650 and 756 cm^-1^), C-O functions (1219, 1095 and 1026 cm^-1^), and cyclopropane C-H (3018 cm^-1^) together with C-H stretch bonds (2916 and 2848 cm^-1^). ^1^H-NMR revealed five singlet methyls at δ_H_ 1.70 (s, Me_27_), 0.94 (s, 6H: Me_18_, Me_30_), 0.87 (s, Me_28_) and 0.78 (s, Me_29_), one secondary methyl group at 0.84, and a pair of doublets in the up-field area (δ_H_ 0.30, *J *= 4.2 Hz and 0.52, *J *= 4.2 Hz), characteristic of cycloartane cyclopropane ring. A double doublet carbinolic proton at δ_H _3.25 (dd, *J*_ax,ax_ = 11.1 , *J*_ax,eq_= 4.5 Hz, H_3_), with reference to its axial and α-orientation, assigned the hydroxyl group as 3*β*-OH. Using HMBCs, the downfield carbinolic proton at δ_H_ 3.99 (t, *J *= 6.3 Hz, H_24_), showed connectivity with a pair of olefinic protons at δ_H_ 4.80 (d, *J *= 1.2 Hz) and 4.90 br-s (each one H), suggesting a terminal methylene. As a whole, the six-degree of unsaturation and the ^13^C-NMR data (Table 1), suggested the presence of a double bond and, therefore, a pentacyclic skeleton. EI-MS fragmentation pattern, supported m/z 355.3018 [C_25_H_39_O]^+^ and 302.2616 [C_21_H_34_O]^+^, typical ions of 4,4’ dimethyl 9,19 cycloesterols (Figure 2). 

The presence of the monounsaturated side chain was confirmed by the *m/z *313.2502 [C_22_H_33_O]^+^, 315 [C_22_H_35_O]^+^ and 297.2587 [C_22_H_33_]^+^. In addition, 381.3153 [M-H_2_O-C_3_H_7_]^+ ^together with 355.3018 [M-H_2_O-C_5_H_9_] ^+^, fragmented due to the elimination of parts of side chain (during a Mc Lafferty process), inferred presence of hydroxyl group in side-chain as is shown in Figure 2 (11). Regarding to these findings and the published data (11), compound 1 was identified as cycloart-25-en-3*β*,24-diol.

HR-EI-MS identified compound 2, as C_31_H_52_O with molecular ion peak at m/z 440.4015 (calc. for C_31_H_52_O: 440.4018, Δ 0.7 ppm). The IR spectrum confirmed absorption of hydroxyl group (3311 cm-1), double bond peak (1640 and 771 cm^-1^), C-O functions (1219 cm^-1^) and C-H stretching at 3020 (cyclopropane ring), 2916 and 2848 cm^-1^. Six degree of unsaturation suggested a double bond (Table 1) and consequently five rings in the molecule. The resonances encompassed thirty-one carbons including seven methyls, twelve methylenes, six methines and six quaternary carbons. ^1^H-NMR revealed five singlet methyls at δ_H_ 1.01 (d, *J *= 3 Hz, Me_27_), 0.99 (d, *J *= 3 Hz, Me_26_), 0.94 (s, 6H: Me_18_, Me_30_ ), 0.88 (s, Me_28_), 0.86 (d, *J *= 6 Hz, Me_21_) and 0.79 (s, Me_29_), a pair of doublets in the up-field area at δ_H_ 0.30 and 0.53 (*J *= 4.25 Hz) indicative of cyclopropane ring characteristic of cycloartanes. A doublet of doublet proton at δ_H_ 3.26 (dd, *J*_ax,ax_ = 11.0 , *J*_ax,eq_= 4.0 Hz, H3), indicative of equilateral β orientated hydroxyl-group, and one pair of olefinic protons δ_H_ 4.64 (d, *J *= 0.5 Hz) and 4.69 (br-s) related to exocyclic terminal methylene. Based on these data, compound 2 was determined as 24-methylene-cycloartan-3*β*-ol (12), confirmed by the EI-MS fragments m/z 425 [M-CH_3_], 407 [425-H_2_O], 315 [M-side chain], 297[315-H_2_O], 300[C_22_H_36_] and 175 [300-sidechain].

Compound 3 obtained by the acetylation of 1, was identified as 3*β*, 24-O-diacetyl-cycloart-25-en through EI-MS molecular ion peak m/z 526 [M]^+^, 466 [M-CH_3_COOH]^+^, 423[466-CH_3_CO], 406[466-CH_3_COOH]^+^ and ^1^H-NMR spectrum. The IR spectrum supported absorptions at 3020 (cyclopropane ring), 2936, 2868, 1736 (esteric carbonyl), 1650, 1456, 1373, 1244, 1026 and 758 cm^-1^ without hydroxyl group peak at [3500-3300 cm^-1^]. Likewise, the structure of compound 4 after acetylation of 2, was confirmed as 3*β*-O-acetyl-24-methylene-cycloartan on the bases of EI-MS molecular ion peak m/z 482 [M]^+^ and 422 [M-COOH]^+^, IR and NMR spectra. IR spectrum showed absorption at 3018 (Cyclopropane ring), 2916, 2848, 1736 (Esteric carbonyl), 1642, 1456, 1373, 1244, 1026 and 758 cm^-1^ without any peak at hydroxyl area [3600-3200 cm^-1^] and the signals of δ_H_ 4.64 (d, *J *= 0.5 Hz) and 4.69 br-s, each one H related to external methylene, 3.26 (dd, *J*_ax,ax_ = 11.0 , *J*_ax,eq_= 4.0 Hz, H3) geminal to oxygenated carbon, 2.03 (s, acetate methyl), 1.01 (d, *J *= 3 Hz, Me_27_), 0.99 (d, *J *= 3 Hz, Me_26_), 0.94 (s, 6H:Me_18_, Me_30_ ), 0.88 (s, Me_28_), 0.86 (d, *J *= 6 Hz, Me_21_), 0.79 (s, Me_29_) together with 0.30 (d, 4.25 Hz) and 0.53 (*J *= 4.25 Hz) of cyclopropane ring were observed in ^1^H-NMR spectrum.

*Proliferation assay*

**Figure 3:**
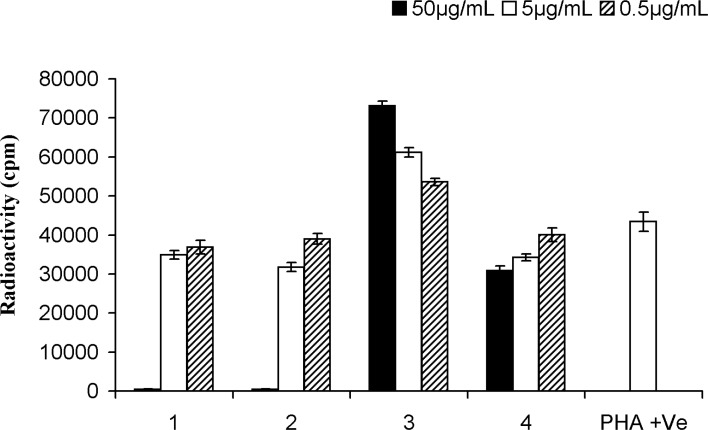
Proliferation assay on peripheral blood lymphocytes of cycloartanes in *Euphorbia aellenii. *cycloart-25-en-3*β*,24-diol (1), 24-methylene-cycloartan-3*β*-ol (2), 3*β*, 24-O-diacetyl-cycloart-25-en (3), 3*β*-O-acetyl-24-methylene-cycloartan (4), T-cells were stimulated by phytohemagglutinin (PHA) in the presence of three different concentrations of compounds. Significance differences between the means of compounds as compared to the control (PHA +ve) were calculated by using one-way ANOVA at p = < 0.05

The anti-proliferation effect of the test compounds was determined by measuring the PHA-induced T-cell proliferation by determining radioactive thymidine incorporation. Comparison of pasitive, negative controls were included to assiss the activity of test compounds. cycloart-25-ene-3*β*, 24-diol (1) showed dose-dependent decrease in lymphocyte proliferation with IC_50_: 12.1 ± 0.6 μg/mL. This result was in conformity with another study by Smith-Kielland (14) which showed cytotoxic activity against Ehrlich ascites tumor cells in mice. Likewise, 24-methylene-cycloartan-3*β*-ol (2), presented dose dependent inhibitory effect with IC50: 10.4 ± 0.1 μg/mL agreed with other published data supporting pain-relieving activity, and anti-inflammatory effect by TPA-induced ear oedema in mice (15). Masking free OH groups of 1 and 2 by acetylation, anti-proliferative effect decreased significantly (IC_50_ > 50 μg/mL). On the other hand, in the case of 3 with two acetyloxy groups (3-OAc and 24-OAc), proliferation of PBLs increased by 23-25% at the low concentration (0.5 μg/mL) in comparison with PHA (5 μg/mL) as positive control. However of the higher concentration 50 and 5 μg mL− 1 of 66-70% and 36-39% increas in prolification were absorb. These results suggested that the proliferation stimulatory activity on PBLs is related to the presence of 24-OAc function while anti-proliferation effect induced by free 3-OH group (Figure 3).

*Docking results*

**Figure 4:**
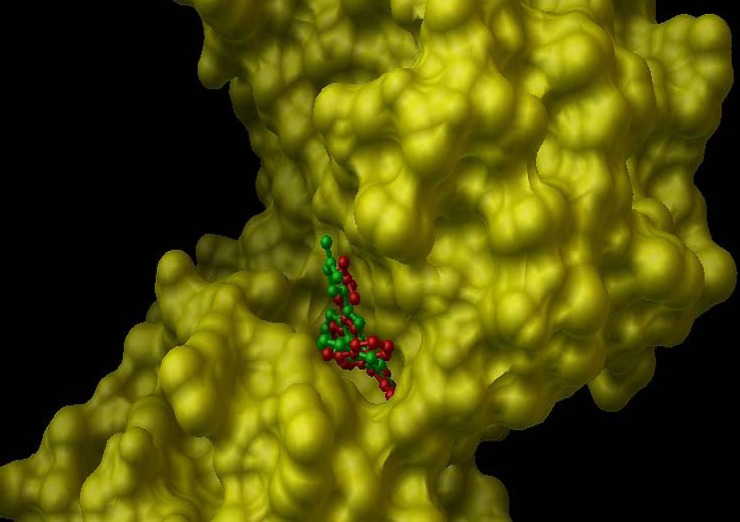
Internal validation phase result. PKC active site structure rendered as solvent-excluded surface (SES) and conformational comparsion of bisindolylmaleimide from crystal structure (green structure) with that from AutoDock model (red structure).

**Figure 5:**
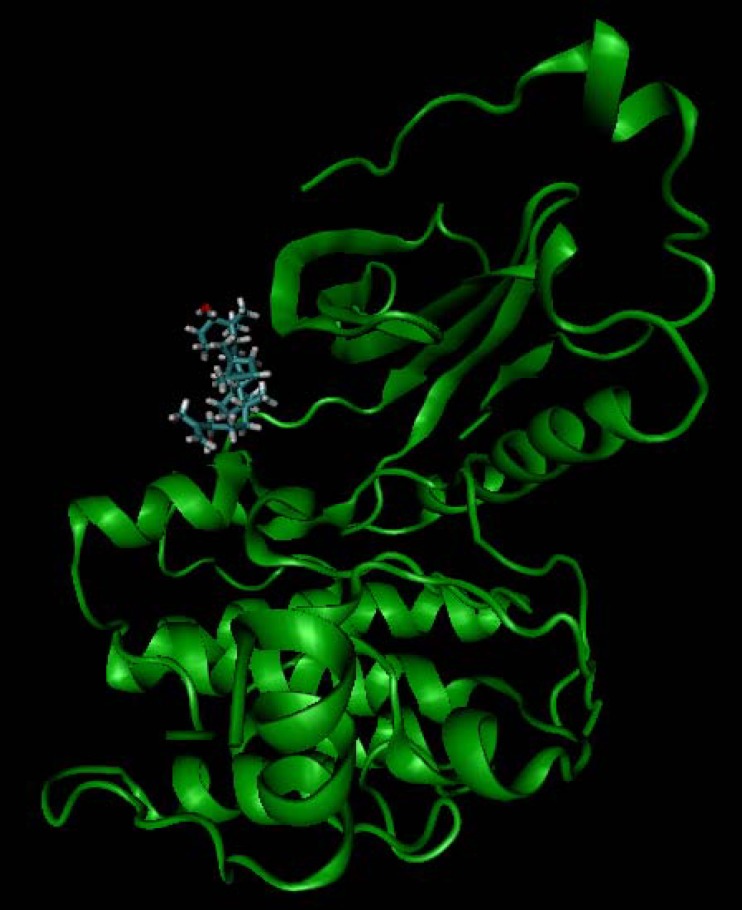
Docking simulation result of cycloart-25-en-3β,24-diol (1).

In the internal validation phase of docking, bis(indolyl) maleimide was docked onto the PKC and the lowest energy pose for docking is shown in Figure 4. Superimposing the experimental and predicted conformations, the RMSD was achieved as 1.02 A°, considered as a successful docking (16) of such ligands with PKC. Thereafter, the proposed mechanism of the action was validated by docking the compounds (1-4) in the binding site (Figure 5). All of four compounds tended to accept similar orientations, and docked into the active site of PKC. In compounds (1-2) forming hydrogen-bond interactions between 3-OH and Pro-532, could explain the antiproliferative effect of T-cell derived PKC *in-vitro. *Therefore, based on this structure-function study, the presence of 3-OH could be correlated with the ability to deactivate PKC and inhibited T-cell proliferation. Likewise, hydrogen bond interaction between 24-O-acetyl group (3) and Asp-330 of PKC active site could be responsible for induction of lymphocyte proliferation derived PKC *in-vitro*. 

## Conclusion

For the first time Cycloart-25-en-3*β*,24-diol (in high quantity) and 24-methylene-cycloartan-3*β*-ol could be isolated from *Euphorbia aellenii***. **Their immunomodulatory effects suggested that the proliferation stimulatory activity on PBLs is related to the presence of 24-OAc function while anti-proliferation effect induced by free 3-OH group. The SAR studies on PKC confirmed these results and provided further support for the role of PKC in transduction of activation signals in T-cells. 
